# Gram stain to the rescue: a case report of cerebral phaeohyphomycosis by *Cladophialophora bantiana* in an immunocompetent 24-year-old

**DOI:** 10.1186/s12879-021-07008-4

**Published:** 2022-01-04

**Authors:** Perceus Mody, Paul Wada, Karen C. Bloch, Michail S. Lionakis, Katie D. White, Alexander S. Maris, Tonya Snyder, Jennifer Steinhauer, Romney Humphries

**Affiliations:** 1 Department of Pathology, Immunology and Microbiology, Vanderbilt University Medical Center, 1301 Medical Center Dr., TVC 4524, Nashville, TN 37232 USA; 2 Department of Medicine, Division of Infectious Diseases, Vanderbilt University Medical Center, Nashville, TN USA; 3 Fungal Pathogenesis Section, Laboratory of Clinical Immunology and Microbiology, National Institute of Allergy and Infectious Diseases, Annapolis, MD USA; 4 Division of Intramural Research, National Institute of Allergy and Infectious Diseases, Annapolis, MD USA

**Keywords:** *Cladophialophora bantiana*, Brain abscess, Dematiaceous mold, Melanized fungi, Cerebral phaeohyphomycosis, Case report

## Abstract

**Background:**

Fungal brain abscesses in immunocompetent patients are exceedingly rare. *Cladophialophora bantiana* is the most common cause of cerebral phaeohyphomycosis, a dematiaceous mold. Radiological presentation can mimic other disease states, with diagnosis through surgical aspiration and growth of melanized fungi in culture. Exposure is often unknown, with delayed presentation and diagnosis.

**Case presentation:**

We present a case of cerebral phaeohyphomycosis in a 24-year-old with no underlying conditions or risk factors for disease. He developed upper respiratory symptoms, fevers, and headaches over the course of 2 months. On admission, he underwent brain MRI which demonstrated three parietotemporal rim-enhancing lesions. Stereotactic aspiration revealed a dematiaceous mold on staining and the patient was treated with liposomal amphotericin B, 5-flucytosine, and posaconazole prior to culture confirmation. He ultimately required surgical excision of the brain abscesses and prolonged course of antifungal therapy, with clinical improvement.

**Conclusions:**

Culture remains the gold standard for diagnosis of infection. Distinct microbiologic findings can aid in identification and guide antimicrobial therapy. While little guidance exists on treatment, patients have had favorable outcomes with surgery and combination antifungal therapy. In improving awareness, clinicians may accurately diagnose disease and initiate appropriate therapy in a more timely manner.

## Background

Primary cerebral phaeohyphomycosis is a rare infection caused by brown-black pigmented fungi, namely dematiaceous (melanized) molds [1]. It was first described in 1974 as tissue invasion by pigmented septate hyphae [2]. Of note, more than 150 species and 70 genera have been implicated in a variety of human diseases [3]. They are rarely involved in central nervous system (CNS) infections, however they have a proclivity for immunocompetent hosts and those with inherited CARD9 deficiency [4, 5]. The most commonly cultured melanized fungi include *Cladophialophora*, *Rhinocladiella, Curvularia*, *Verruconis*, *Exophiala*, *Veronaea* and *Fonsecaea* [6–10]. In a retrospective review of 101 cases of primary CNS phaeohyphomycosis, *Cladophialophora bantiana* was by far the most common species (48%) worldwide, followed by *Rhinocladiella mackenziei* (13%) which was more common in the Middle East [1, 11, 12].

*Cladophialophora bantiana* is a melanized, highly neurotropic fungus present in soil. It prefers a warmer climate with high humidity and has a wide geographic distribution (Asia, North and South America, Europe, and Africa) [11, 13, 14]. *Cladophialophora bantiana* virulence mechanisms are not clearly understood. A possible mechanism involves melanin production that interferes with microglial recognition, scavenging free radicals and hence preventing eradication of fungi from brain parenchyma [15, 16]. In animal models, a common route of infection is through inhalation of airborne conidia [17], although lung infection with cerebral involvement is rarely documented [11]. Demographic data from reported cases demonstrate higher rates in men, residents from rural areas, and occupations such as farmers, agricultural workers, florists, gardeners, fruit workers and coal miners [1, 11, 18–20]. More than half of infections occur in patients with no known immunosuppression [1, 7]. Although other agents of cerebral phaeohyphomycosis have been described in patients with CARD9 deficiency, there are no reports to date of *C. bantiana* infections associated with this primary immunodeficiency [5]. Mutations in CARD9 affect innate immunity through alterations in signaling and recognition of the fungal cell wall [21].

Early diagnosis and treatment of dematiaceous molds can be challenging, with radiological features that mimic high grade gliomas, lymphomas, or tuberculomas [14, 22–25]. In one review of *C. bantiana* brain abscesses, diagnosis was delayed a mean 115 days from development of symptoms [19].

## Case presentation

A 24-year-old man with no significant past medical history and on no medications presented to the emergency department with three weeks of headaches. One month prior to presentation he developed sinus pain, subjective fevers, nasal congestion, and ear fullness lasting for one week. He received an unknown course of antibiotics at the time. The symptoms improved; however, he noticed a new, constant headache with associated nausea, vomiting, blurry vision, and night sweats. He worked as a welder with prior employment in the coal industry. His hobbies included hunting and fishing and he reported significant time spent outdoors. Over the summer, he aerated his dirt basement after a plumbing leak.

**Fig. 1:**
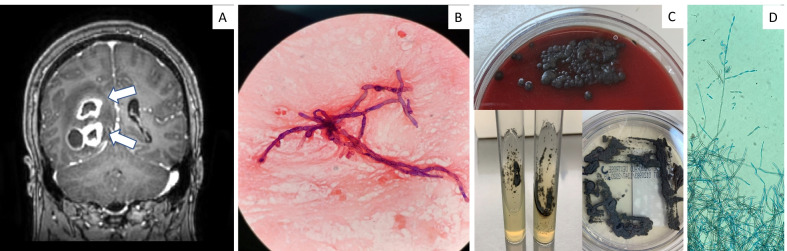
**A** T1-weighted contrast-enhanced magnetic resonance image (MRI): coronal section through the posterior cranial fossa shows multiple rim-enhancing abscesses (arrows pointed) in the parieto-temporal lobes. **B** Gram-stain showing septate hyphae with sparse branching, under × 1000. **C** Olive-black velvety colonies of *C. bantiana* growing on Sheep Blood Agar, Sabouraud’s Slant and Sabouraud’s Plate. **D** Tape preparation of *C. bantiana*, stained with lactophenol cotton blue, showing melanized septate hyphal elements, under × 400

On presentation, he was afebrile with normal vital signs and a non-focal physical exam. He had a white blood cell count of 10.4 × 10^3^/μL (normal range 3.9–10.7 × 10^3^/μL) with 0% eosinophils and 80% neutrophils (absolute neutrophil count of 8.37 × 10^3^/μL). Erythrocyte sedimentation rate was 22 mm/h (normal range 1–33 mm/h) and C-reactive protein was 3.1 mg/L (normal range 0–5 mg/L). Computed tomography (CT) of the head was notable for three right parietotemporal masses with 9 mm midline shift and significant edema; sinus CT ruled out rhino-orbital involvement. He was started on dexamethasone with subsequent magnetic resonance imaging (MRI) confirming three irregular rim-enhancing lesions (Fig. 1A). He was taken to the operating room where he underwent stereotactic abscess evacuation with aspiration of purulent material. This was sent for bacterial, fungal and mycobacterial cultures and histopathological analysis. Microscopic examination of the abscesses revealed septate hyphal elements on the Gram stain (Fig. 1B).

Based on the gram stain result, he was started on liposomal amphotericin B, five milligrams per kilogram intravenously once daily. By day 6, fungal and bacterial cultures had grown a dematiaceous mold with velvety, olivaceous black colonies (Fig. 1C) at 30 °C and 35 °C. Tape preparation of colonies with lactophenol cotton blue revealed long brown septate hyphae with sparsely branched wavy chains of smooth conidia (Fig. 1D). 5-Flucytosine 25 mg per kilogram by mouth four times daily and posaconazole 300 mg by mouth twice daily were added. Serum galactomannan antigen and (1,3)-β-d-glucan serum tests returned negative, while baseline positron emission tomography (PET) CT showed known brain abscesses with a few small 5–6 mm lower lobe pulmonary nodules. Repeat MRI brain was obtained two weeks after the original surgery and demonstrated progression of abscesses and midline shift. He underwent operative debulking with resultant homonymous hemianopsia, but otherwise tolerated treatment well. He completed a 6-week course of liposomal amphotericin B and has remained on 5-flucytosine and posaconazole since discharge.

## Discussion and conclusions

Cerebral biopsy with microbiological culture and histological analysis remains the gold standard for diagnosis of brain abscesses. Gram stain of this patient’s parietal abscess allowed for visualization of septate hyphae with growth in media in 6 days. DNA sequencing and susceptibility results returned after 13 days of isolate submission to the reference laboratory, University of Texas Health Science Center at San Antonio. Sequencing targets included ITS, TUB and TEF, with susceptibility results tabulated in Table 1. It can be challenging to identify *C. bantiana* phenotypically if mycology is not routinely performed at the home institution. This may lead to further delays in diagnosis since the organism must be sent out for reference identification. *Cladophialophora bantiana* forms olivaceous black colonies with a velvety texture and can grow at higher temperatures (42 °C). Microscopically, its short chaining septate hyphae and smooth conidia are similar to *Cladosporium spp*, however it lacks conidiophores [3, 26].

**Table 1:** *Badali, H., et al., Use of amplified fragment length polymorphism to identify 42 Cladophialophora strains related to cerebral phaeohyphomycosis with in vitro antifungal susceptibility. J Clin Microbiol, 2010.  **48**(7): p. 2350–6

Antifungals	mg/L	In-vitrosusceptibility MIC range (n = 37, mg/L)*	Interpretation**
Amphotericin B (AMB)	4	0.12–2.0	No established breakpoints
5-Fluorocytosine (5-FC)	0.25	Not listed	No established breakpoints
Posaconazole (POS)	< = 0.03	0.01–0.25	No established breakpoints
Voriconazole (VORI)	0.125	0.12–4.0	No established breakpoints

^**^Methodology: CLSI M38

There is relatively little guidance on treatment of CNS phaeohyphomycosis in the literature [27]. In the few cases reported, surgical intervention with combination antifungal therapy is warranted [3, 19, 27]. The optimal duration of treatment is not clearly defined but antifungal therapy is typically given for several weeks to months or longer [28]. Despite aggressive intervention involving wide excision of infected parenchymal tissue, the mortality rate remains over 70%, particularly in immunosuppressed patients. Furthermore, prognosis is worse in patients with multiple lesions [1, 16, 19, 20, 29, 30]. Among available antifungal treatments for CNS phaeohyphomycosis, 5-flucytosine and voriconazole achieve a high cerebrospinal fluid concentration with their low molecular weight, low to intermediate lipophilicity, and lower protein binding [27, 31]. Liposomal amphotericin B and posaconazole provide less CNS penetration as relatively larger molecules, however they are often used as part of combination antifungal therapy [32–34]. Prolonged voriconazole use may be limited by its propensity to cause skin cancer, a known association in solid organ transplant recipients, and fluorosis [6, 27, 35]. Posaconazole may be an appropriate alternative for long-term maintenance and suppressive therapy with excellent in vitro activity [4].

Several barriers to treatment can limit favorable outcomes. For one, a scarcity of cases complicate efforts to compare treatments, with therapeutic recommendations based on in vitro data [36] and expert opinion only. Murine models suggest that residual mold may persist in brain tissue even after extended antifungal treatment—for this reason, patients often require indefinite suppressive therapy [6, 27, 37]. Finally, long hospital stays are often necessary for invasive surgical debridement measures and administration of liposomal amphotericin B in a closely monitored setting.

*Cladophialophora bantiana* CNS infections are exceedingly uncommon in North America. Despite improved understanding of the disease and accessibility of therapeutic options, prognosis remains poor. Highlighting documented cases in the literature can increase awareness, with gradual progress towards earlier detection and standardized treatment. Nevertheless, the relationship between types of diagnostic methods and patient outcomes should be explored further. Gram stain as well as accurate and timely microbiological analysis provided direct benefit for our patient, but novel approaches like next-generation sequencing could play a complementary role in identification of disease and potentially reduce time from symptom onset to diagnosis in the future.

## Data Availability

All data generated or analyzed during this study are included in this manuscript.

## Abbreviations

CT: Computed tomography
PET: Positron emission tomography
MRI: Magnetic resonance imaging
CNS: Central nervous system
DNA: Deoxyribonucleic acid
CARD9: Caspase recruitment domain-containing protein 9
ITS: Internal transcribed spacer
TUB: Tubby protein homolog
TEF: Thyrotroph embryonic factor
